# *Paenibacillus* spp. isolated from human and environmental samples in Spain: detection of 11 new species

**DOI:** 10.1016/j.nmni.2017.05.006

**Published:** 2017-05-24

**Authors:** J.A. Sáez-Nieto, M.J. Medina-Pascual, G. Carrasco, N. Garrido, M.A. Fernandez-Torres, P. Villalón, S. Valdezate

**Affiliations:** Taxonomy Laboratory, Bacteriology Area, Centro Nacional de Microbiología, Instituto de Salud Carlos III, Madrid, Spain

**Keywords:** Clinical samples, environmental isolates, new species, *Paenibacillus*, polyphasic taxonomy

## Abstract

One hundred thirty-six isolates, 88 human and 48 environmental, that met the requirements to belong to the genus *Paenibacillus* were identified using a polyphasic taxonomic approach known as 16S rRNA plus phenotypic traits. Thirty-seven *Paenibacillus* species were identified; some had not been previously reported from clinical samples. The main species were *P. pabuli* (13 isolates), *P. provencensis* (11), *P. phoenicis* (9) and *P. lautus* (8). *P. pabuli* (11/13) and *P. provencensis* (8/11) were mainly environmental isolates, while *P. phoenicis* (9/9) and *P. lautus* (6/8) were mainly human isolates. Despite the difficulties in assigning to human *Paenibacillus* isolates a role as a pathogen or contaminant, here 25% of the isolates were involved in true infections, especially in those cases that affected abscesses, wound exudates, ocular infections and diverse fluids. In addition, 15 isolates were identified as 11 *‘Candidatus’* to a new species, all of them from human specimens except one that was obtained from laboratory air*.* The antimicrobial susceptibility testing showed 95.6% of isolates were resistant to ampicillin, 44% were resistant to cotrimoxazole, 20 to 30% were resistant to cefotaxime and vancomycin and 13% were resistant to rifampicin and erythromycin.

## Introduction

The genus *Paenibacillus* is characterized as rod-shaped Gram-positive or Gram-variable endospore forming aerobic or facultatively anaerobic bacteria. Originally derived from a *Bacillus,* group 3 was proposed by Ash et al. in 1993 [1] on the basis of comparative 16S RNA gene sequence analysis. *Paenibacillus* species have been isolated from a variety of sources including soil, fresh and salt water, sewage, sediments, caves, humus, compost, rhizosphere, food, plants, insect larvae and clinical samples. The genus contains 211 recognized species [2] (http://www.bacterio.net/paenibacillus.html). Only 22 species has been reported from human clinical samples: *P. alvei, P. faecis, P. ihumii, P. konsidensis, P. larvae, P. lautus, P. macerans, P. massiliensis, P. pasadenensis, P. polymyxa, P. provencensis, P. residui, P. sanguinis, P. sputi, P. thiaminolyticus, P. timonensis, P. turicensis, P. urinalis* and *P. vulneris* [3], [4], [5], [6], [7], [8], [9], [10], [11], [12], [13], [14], [15], [16], [17], [18], as well as two species still officially not recognized (*P. honkongensis* and *P. dakarensis*) [19], [20] and recently *P. antibioticophila* [21]. Those strains were recognized as responsible for true infections or transient infections colonizing blood and other human sources, or as a possible contamination occurring during the process of obtaining the sample.

In the present study, we identified *Paenibacillus* species isolated from clinical specimens and environmental samples received from hospital laboratories and public health centres using a polyphasic taxonomic approach.

## Materials and methods

### Bacterial strains

One hundred thirty-six *Paenibacillus* isolates from 44 hospital and public health laboratories from 28 Spanish provinces were sent to our laboratory for identification between 1999 and 2015. The distribution of the 88 human samples was as follows: blood (56 isolates), wounds and skin abscesses (10), ascitic fluid (5), cerebrospinal fluid (3), joint fluid (3), eye (3), sputum (2) and one each of brain abscess, pericardium, vascular catheter, urine and lung. Meanwhile, the 48 environmental samples included control laboratory workers' gloves (18 isolates), air (10), surfaces (6), water (1), biological products and drugs (8) and reagents (4). One strain was isolated from sea lion faeces (Antarctica).

The preliminary identification of isolates reported by laboratories was varied: *Bacillus* spp., *Penibacillus* spp., Gram-variable bacilli, Gram-negative bacilli and sporulated Gram-positive bacilli or nonfermenting Gram-negative bacilli.

### Identification of *Paenibacillus* species

All received isolates were plated on Columbia agar with 5% defibrinated sheep's blood for 24 to 48 hours in aerobic conditions at 37°C. Gram stain showed Gram-positive bacilli or Gram-variable sporulated bacilli. The colonial morphology varied and included shiny, smooth colonies as well as highly mucous and irregular forms. Chromosomal extraction was undertaken by boiling the cultures.

### 16S RNA gene sequencing and analysis

For partial sequencing of the 16S rRNA gene of the strains, two types of primers were used. The first set was used in all strains (fragment of ∼1400 bp) and was described by Drancourt et al. [22]. The other, described by Shida et al. [23] (900–1000 pb), was more specific to the genus *Paenibacillus* and was used when the first set primers did not clearly differentiate between species. Purification, sequencing and analysis of the sequences were performed according to strategies previously described in our laboratory, and phylogenetic trees were constructed using the neighbour-joining method described previously [24]. The 16S fragments sequenced for each isolate were compared to sequences in the GenBank database and identified by BLAST 2.2.10 (http://www.ncbi.nlm.nih.gov/BLAST). A similarity score of ≥99.0% between the 16S rRNA sequence and database sequence was deemed to indicate that the isolate belonged to the same species.

### Phenotypic identification

When the reference standard method for identification, 16S rRNA sequencing, did not differentiate among closely related species, two type of panels were used: API 20E, API 20NE, API CHB, API Zym panels (bioMerieux, Marcy-l’Etoile, France) and Biolog GP2 microplates (Biolog, Hayward, CA, USA) to study the oxidation reduction of different substrates.

Thus, to differentiate isolates of related species, we studied, first, catalase and oxidase growth at 50°C and fermentation patterns of various sugars (API CHB) for *P. massiliensis* vs. *P. timonensis;* second, the growth in anaerobiosis, at 50°C and with 5% of ClNa, with the Voges-Proskauer test (API 20E), to distinguish *P. cineris, P. rhizospherae, P. flavisporus;* and third, the tests contained in panels API CHB panel and BIOLOG GP2 (redox reactions) to differentiate *P. pabuli, P . xylanolyticus, P. tundrae, P. taichungensis* and *P. xylanexedens.*

### Phylogenetic tree based on 16S rRNA analysis of *Paenibacillus* spp. sequences

Sequences were assembled using SeqMan software (DNAStar, Madison, WI, USA). The sequence lengths were adjusted to match the length of the shortest sequence of each species and were aligned using the ClustalW algorithm (http://www.ebi.ac.uk/Tools/clustalw2/index.html). A phylogenetic assessment of each species was undertaken using MEGA 4.1 software, and phylogenetic trees were constructed using the neighbour-joining, with bootstrap analyses based on 1000 resamplings. Branches corresponding to partitions that were reproduced in <50% of bootstrap replicates were collapsed.

### Antimicrobial susceptibility

Antimicrobial susceptibility testing to eight antimicrobials (ampicillin, cefotaxime, erythromycin, minocycline gentamicin, cotrimoxazole, rifampicin and vancomycin) was determined by Etest on Mueller-Hinton agar with 5% of defibrinated sheep's blood, incubated in aerobiosis at 37°C and read after 48 hours. The interpretative criteria described for *Bacillus* strains were adopted [25] because no Clinical and Laboratory Standards Institute breakpoints are available for *Paenibacillus.*

## Results

For the identification of the *Paenibacillus* species, the partial sequencing of the 16S rRNA gene was effective. However, some of them were not well discriminated with this method. For the resolution of these cases, different panels of phenotypic tests were used. Different algorithms for a number of phenotypic tests were used to differentiate the identities of the strains that did not differ by the sequencing method.

Table 1 shows the identification and the source of isolation of 136 strains of *Paenibacillus* submitted to our reference laboratory. The strains belonged to 35 different species. Five strains were not assigned to any species, and 15 strains are part of 11 *‘Candidatus’* to new species. The species most frequently identified were *P. pabuli, P. provencensis, P. phoenicis* and *P. lautus.* The first two species were mainly isolated from environmental samples: *P. pabuli* (11/13) and *P. provencensis* (8/11). *P. phoenicis* (9/9) and *P. lautus* (6/8) were isolated from human sources. Of the 37 species identified plus the 11 *‘Candidatus,’* 12 species were isolated from both clinical and environmental samples. On the other hand, 19 species and ten *‘Candidatus’* were found only in human samples, and five species were only isolated from environmental samples (*P. assamensis, P. barcinonensis, P. graminis, P. xylanexedens* and *‘Candidatus P. aerius’*). According to the previous literature, of the 22 *Paenibacillus* species that were isolated from human sites, in our study we detected 11 species (*P. alvei, P. larvae, P. lautus, P. macerans, P. massiliensis, P. polymyxa, P. provencensis, P. thiaminolyticus, P. timonensis, P. turicensis* and *P. antibioticophila*). Likewise, in our study we found 23 species not previously isolated from human samples (Table 1).

**Table 1:** One hundred thirty-six *Paenibacillus* spp. isolates from human and environmental sources (1999–2015) in Spain

*Paenibacillus* spp. (no. of isolates)	Human source (no. of isolates)	Environmental source (no. of isolates)
*P. alvei* (1)	Synovial fluid (1)	—
*P. amylolyticus* (6)	Blood (1)	Gloves^a^ (3), air (2)
*P. anaericanus* (1)	Blood (1)	—
*P. antibioticophila* (1)	Brain abscess (1)	
*P. apiarius* (1)	Blood (1)	—
*P. assamensis* (1)	—	Gloves^a^ (1)
*P. barcinonensis* (3)	—	Gloves^a^ (1), air (1), biological product (1)
*P. barengoltzii* (3)	Ascetic fluid (2), synovial fluid (1)	—
*P. campinansensis* (2)	Blood (2)	—
*P. cineris* (2)	Sputum (1)	Surface (1)
*P. ginsengarvi* (1)	Blood (1)	—
*P. ginsengihumi* (5)	Blood (2), cerebrospinal fluid (1), eye (1)	Gloves^a^ (1)
*P. glucanolyticus* (6)	Blood (2), abscess (2)	Gloves^a^ (1), surface (1)
*P. graminis* (1)	—	Air (1)
*P. humicus* (1)	Blood (1)	—
*P. illinoisensis* (1)	Abscess (1)	Air (1)
*P. lactis* (1)	Blood (1)	—
*P. larvae* (2)	Wound (1), sputum (1)	—
*P. lautus* (8)	Blood (3), abscess (1), wound (2)	Gloves^a^ (1), biological product (1)
*P. macerans* (6)	Blood (4), wound (1), synovial fluid (1)	—
*P. massiliensis* (2)	Blood (1)	Culture medium (1)
*P. motobuensis* (1)	Ascetic fluid (1)	—
*P. naphtalenovorans* (2)	Blood (2)	—
*P. odorifer* (1)	Blood (1)	—
*P. pabuli (13)*	Blood (1), cerebrospinal fluid (1)	Gloves^a^ (5), surface (2), alcohol (1), solution (1), air (1), Antarctic sea lion (1)
*P. phoenicis* (9)	Blood (8), cerebrospinal fluid (1)	—
*P. polymixa* (2)	Blood (2)	—
*P. provencensis (11)*	Blood (2), synovial fluid (1)	Gloves^a^ (4), biological product (1), air (3)
*P. pueri* (1)	Blood (1)	—
*P. residui* (1)	Blood (1)	—
*P. stellifer* (1)	Blood (1)	—
*P. thiaminolyticus* (5)	Blood (1), vitreous humor (1)	Biological product (3)
*P. timonensis* (5)	Blood (3), ascetic fluid (1)	Biological product (1)
*P. turicensis* (1)	Blood (1)	—
*P. vini* (1)	Blood (1)	—
*P. xylanolyticus* (4)	Lung biopsy (1)	Gloves,^a^ water, solution (3)
*P. xylanexedens* (2)	—	Surface (2)
*Paenibacillus* spp. (5)	Blood (2), urine (1), wound (1)	Biological product (1)
*‘Candidatus'*^b^		
*‘P. hispaniensis’* (4)	Blood (3), ascetic fluid (1)	—
*‘P. castillanus’* (2)	Blood (1), wound (1)	—
*‘P. aerius’* (1)	–	Air (1)
*‘P. guadalajarensis’* (1)	Blood (1)	—
*‘P. hominis’* (1)	Blood (1)	—
*‘P. ilicicola’* (1)	Blood (1)	—
*‘P. infantis’* (1)	Blood (1)	—
*‘P. mageritense’* (1)	Catheter (1)	—
*‘P. pamplonensis’* (1)	Cornea (1)	—
*‘P. pericardicum’* (1)	Pericardium necropsy (1)	—
*‘P. valencianus’* (1)	Blood (1)	—
Total of isolates (*N* = 136)	88	48

a Laboratory workers' gloves (control).

b *‘Candidatus’* GenBank accession numbers: *P. hispaniensis* (KJ469897); *P. castillanus* (KJ469899): *P. aerius* (KJ469902); *P. guadalajarensis* (KJ489420); *P. hominis* (KJ469907); *P. ilicicola* (KJ489419); *P. infantis* (KJ469903); *P. mageritense* (KJ469906); *P. pamplonensis* (KJ469901); *P. pericardicum* (KJ469908); *P. valencianus* (KJ469904).

**Fig. 1:**
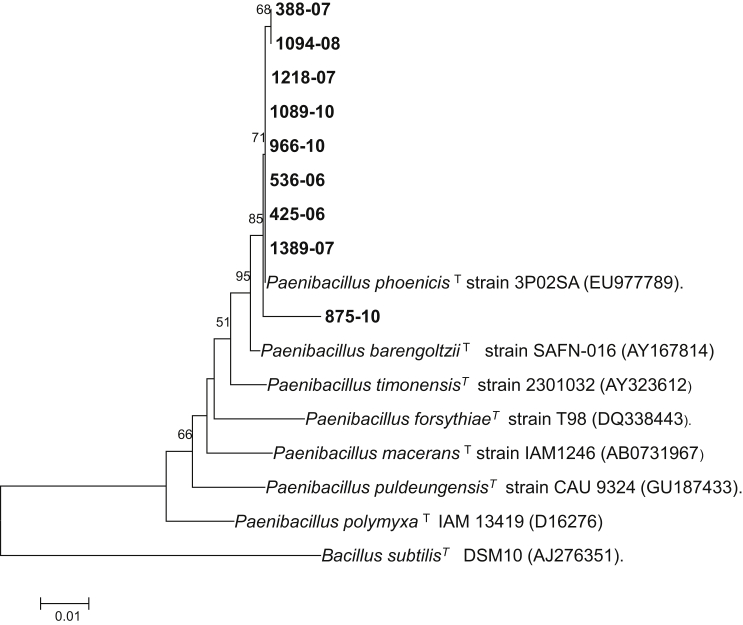
Neighbour-joining phylogenetic tree based on 16S rRNA gene sequence of *Paenibacillus* spp. type (T) and novel (bold type) strains highlighting position of *P. phoenicis* and nine novel strains found in our study relative to other phylogenetically close organisms. Numbers at nodes are percentages of bootstrap values (>50%) obtained by repeating analysis 1000 times to generate majority consensus tree. *Bacillus subtilis* DSM10 was used as outgroup. Scale bar indicates 0.01 nucleotide sequence divergence. Numbers in bold correspond to nine strains identified as *P. phoenicis* in our study.

**Fig. 2:**
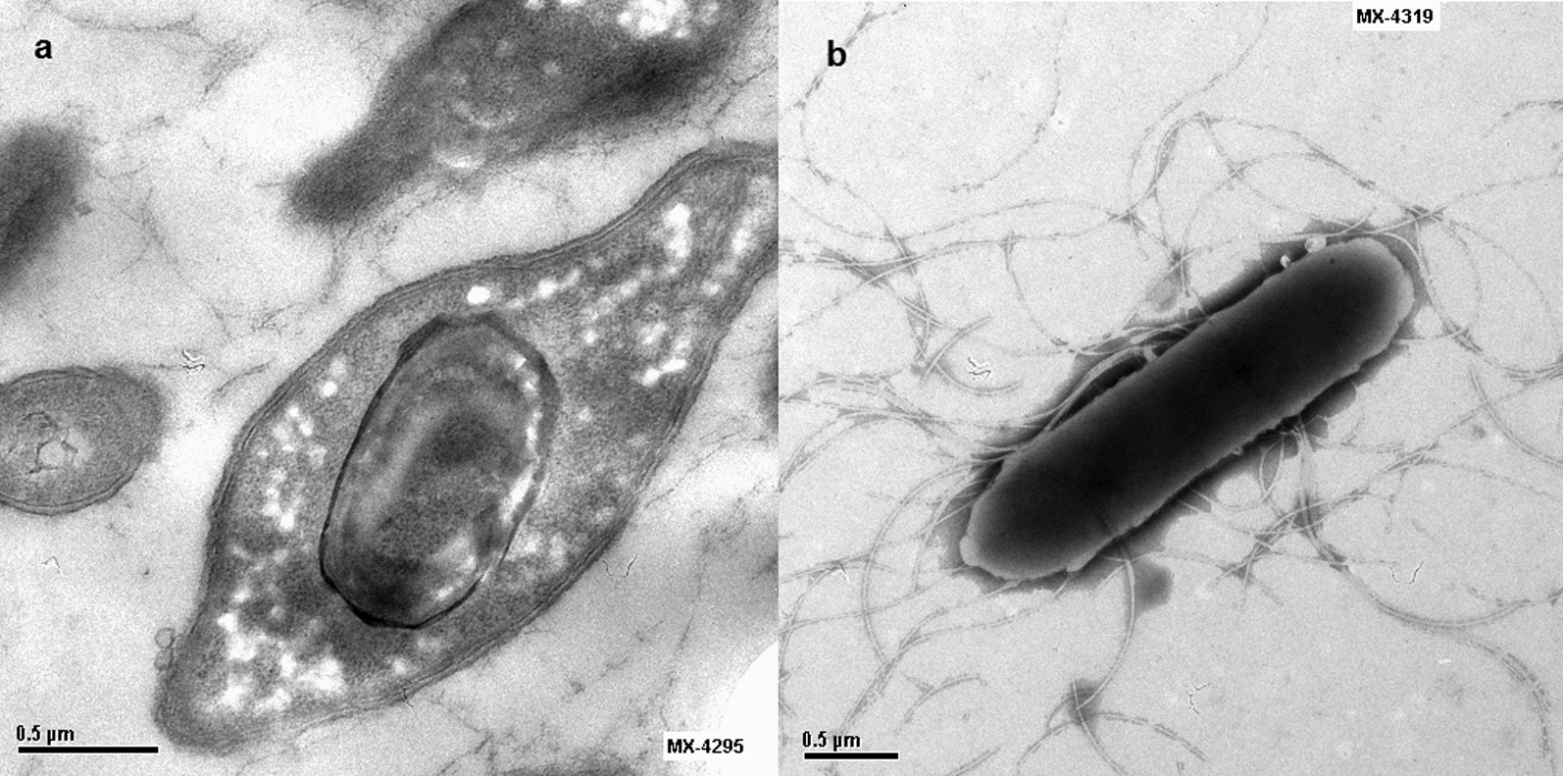
Transmission electron microscopy of *Paenibacillus phoenicis* strain CNM 1389-07 showing (a) oval endospores and (b) peritrichous flagella.

Among the most frequent species mentioned above—*P. pabuli, P. provencensis, P. phoenicis* and *P. lautus—*only *P. phoenicis* had not been isolated from human sources. In our study, between the years 2006 and 2010, nine strains of this species were isolated in three laboratories in different geographic locations. One strain was isolated from cerebrospinal fluid, and the rest were isolated from blood. They were identified by analysis of 16S rRNA gene sequences, and they were confirmed by conventional phenotypic tests studied by API NE and API CHB, coinciding with those described in the *P. phoenicis* type strain. Fig. 1 shows the neighbour-joining phylogenetic tree indicating the position of the isolates of our study and their identity with the type strains of *P. phoenicis* and other closely related species. Fig. 2 shows the typical structure of *Paenibacillus,* with both visualization of flagella and endospores, obtained by electron microscopy.

**Fig. 3:**
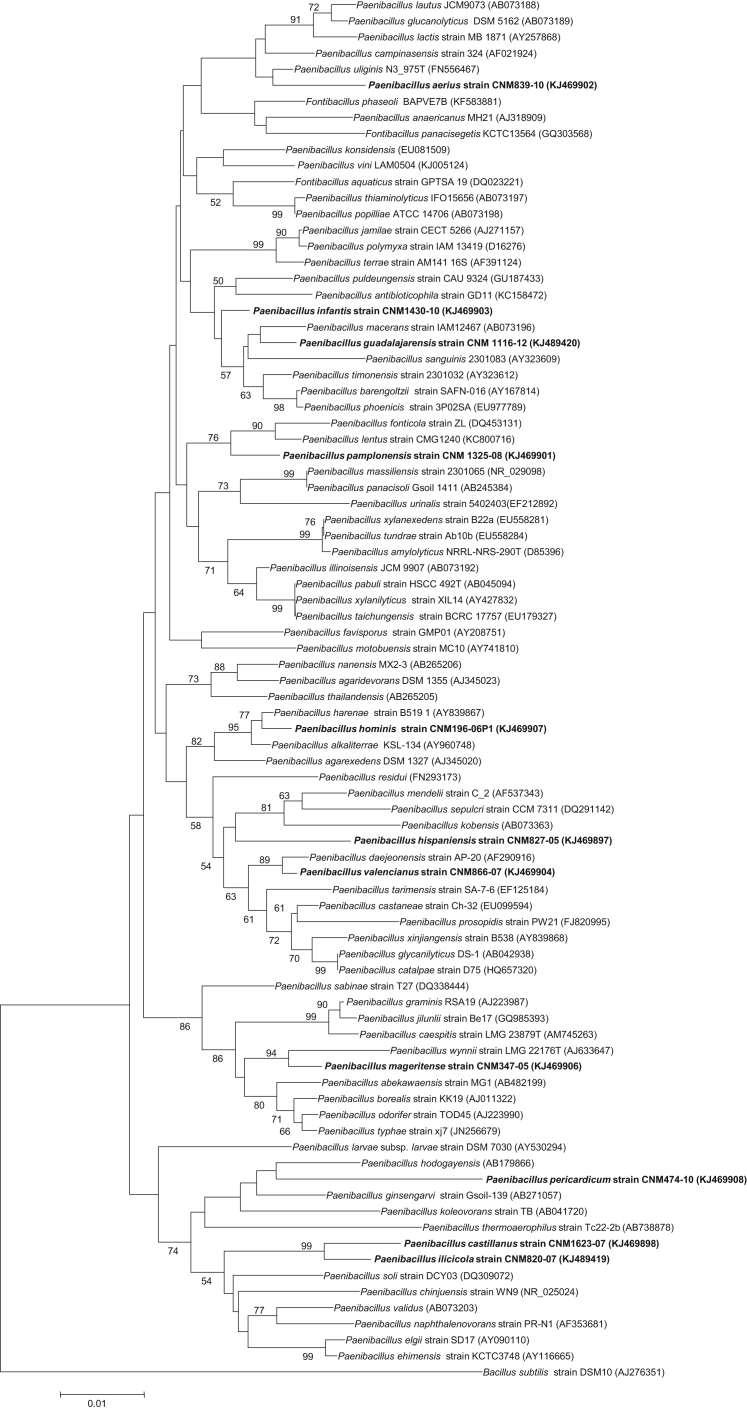
Extended phylogenetic tree according to neighbour-joining method of genus *Paenibacillus* inferred from 16S rRNA gene sequences. Bootstrap values are expressed as percentage of 1000 replications. Numbers at nodes are percentages of bootstrap values (>50%). Scale bar represents 0.01 substitutions per nucleotide position. *Bacillus subtilis* strain DSM10 is used as outgroup. Eleven *‘Candidatus’* species are shown in bold type.

Seventeen isolates were found to meet the phenotypic and genotypic criteria to be *‘Candidatus’* to 11 species of *Paenibacillus.* All but one were isolated from human samples: nine from blood, and one from each ascitic fluid, wound exudate, cornea, pericardial fluid and catheter. One was isolated from an environmental control in a laboratory. In the dendrogram of the species of *Paenibacillus,* we found distribution of *‘Candidatus’* along all the main clusters (Fig. 3, bold).

Table 2 shows the susceptibility of the 136 strains studied against eight antimicrobials. The majority of them (95.6%) were resistant to ampicillin. Only six strains of four species were susceptible: *P. polymyxa* (1 strain), *P. gingensihumi* (2), *Paenibacillus* spp. (2) and *‘Candidatus’ P. pericardicum* (1). Forty-four of the isolates were resistant to cotrimoxazole, 20 to 30% of the strains were resistant to cefotaxime and vancomycin and 13% of isolates were resistant to rifampicin and erythromycin (13%). Half of the isolates showed intermediate minimum inhibitory concentration (MIC) values (1–6 mg/L) against erythromycin; the values were very low or nonexistent for other antibiotics. Only four strains of four different species (*P. alvei, P. timonensis, P. assamensis* and *P. xylanolyticus*) were resistant to gentamicin, and 11 strains of five species were resistant to minocycline (*P. cineris, P. lautus, P. glucanolyticus*, *P. stellifer* and *P. xylanolyticus*). Resistance to rifampicin was found in 18 strains of nine species and 29 strains of 14 species, including two *‘Candidatus’ P. hispaniensis* and *P. valencianus* resistant to vancomycin. Table 3 shows the sensitivity data of the ten most prevalent species. These are homogeneously resistant to ampicillin and sensitive to cefotaxime, gentamicin, rifampicin and vancomycin. All the *P. macerans* isolates were resistant to vancomycin. We found greater variability of sensitivity against erythromycin (especially in those species with intermediate MIC values), except *P. lautus, P. glucanolyticus* and *P. macerans.* Those resistant to cotrimoxazole (*P. pabuli, P. provencensis* and *P. amylolyticus*) were homogeneously resistant. Finally, all *P. glucanolyticus* isolates were resistant to minocycline.

**Table 2:** Overall susceptibility of 136 *Paenibacillus* spp. isolates from human and environmental sources in Spain

Antibiotic	MIC (mg/L)			Breakpoint (mg/L)		Percentage of isolates		
	Range	50^a^	90^a^	Susceptibility	Resistance	Susceptible	Intermediate	Resistant
Ampicillin	0.06 to ≥256	16	≥256	≤0.25	≥0.5	4.4	—	95.6
Cefotaxime	0.03 to ≥256	2	≥256	≤8	≥64	70.6	2.2	27.2
Erythromycin	0.06 to ≥256	1	16	≤0,5	≥8	36.0	50.1	13,9
Minocycline	≤0.016 to 48	0.03	8	≤4	≥16	87.5	4.4	8.1
Gentamicin	≤0.0016 to ≥256	0.5	3	≤4	≥16	95.6	1.5	2.9
Cotrimoxazole	0.003 to ≥32	0.5	≥32	≤2	≥4	56	—	44
Rifampicin	0.003 to ≥32	0.25	8	≤1	≥4	76.4	10.3	13.2
Vancomycin	0.03 to ≥256	3	12	≤4	>4	78.7	—	21.3

MIC, minimum inhibitory concentration.

a MICs at which 50 and 90% of the isolates were inhibited, respectively.

**Table 3:** Antimicrobial susceptibilities of human and environmental isolates belonging to 10 most frequent *Paenibacillus* spp

Antimicrobial (breakpoints)^a^	*Paenibacillus* spp.	No. of isolates	MIC (mg/L)			Categorized isolates, *n* (%)		
			Range	50^b^	90^b^	Susceptible	Intermediate	Resistant
Ampicillin (≤0.25, ≥0.5)	*P. pabuli*	13	1 to >256	>256	>256	—	—	13 (100)
	*P. provencensis*	11	1 to >256	>256	>256	—	—	11 (100)
	*P. phoenicis*	9	0.5 to 32	2	12	—	—	9 (100)
	*P. lautus*	8	2 to >256	48	64	—	—	8 (100)
	*P. amylolyticus*	6	2 to >256	3	>256	—	—	6 (100)
	*P. glucanolyticus*	6	64 to >256	>256	>256	—	—	6 (100)
	*P. macerans*	6	6 to >256	64	>256	—	—	6 (100)
	*P. ginsengihumi*	5	0.06 to 4	0.38	4	3 (60)	—	2 (40)
	*P. thiaminolyticus*	5	3 to 32	4	32	—	—	5 (100)
	*P. timonensis*	5	1 to 64	16	64	—	—	5 (100)
Cefotaxime (≤8, ≥64)	*P. pabuli*	13	>256	>256	>256	13 (100)	—	—
	*P. provencensis*	11	1 to 6	2	4	11 (100)	—	—
	*P. phoenicis*	9	0.06 to 0.5	0.12	0.25	9 (100)	—	—
	*P. lautus*	8	1 to 4	2	2	8 (100)	—	—
	*P. amylolyticus*	6	16 to >256	>256	>256	6 (100)	—	—
	*P. glucanolyticus*	6	0.12 to 4	3	4	6 (100)	—	—
	*P. macerans*	6	0.25 to 2	2	2	6 (100)	—	—
	*P. ginsengihumi*	5	0.19 to 1	0.5	1	5 (100)	—	—
	*P. thiaminolyticus*	5	0.12 to 1	0.12	1	5 (100)	—	—
	*P. timonensis*	5	0.19 to >256	0.38	>256	4 (80)	—	1 (20)
Erythromycin (≤0.5, ≥16)	*P. pabuli*	13	0.06 to 2	0.12	1	11 (85)	2 (15)	—
	*P. provencensis*	11	1 to 2	1.5	2	—	11 (100)	—
	*P. phoenicis*	9	1 to 4	2	2	—	9 (100)	—
	*P. lautus*	8	1 to >256	12	>256	—	2 (25)	—
	*P. amylolyticus*	6	0.06 to 2	1	2	2 (33)	4 (67)	—
	*P. glucanolyticus*	6	0.5	0.5	0.5	6 (100)	—	—
	*P. macerans*	6	0.03 to 0.25	0.06	0.12	6 (100)	—	—
	*P. ginsengihumi*	5	1 to 2	1	2	—	5 (100)	—
	*P. thiaminolyticus*	5	0.25 to 12	0.5	12	1 (20)	3 (60)	1 (20)
	*P. timonensis*	5	1.5 to 2	2	2	—	5 (100)	—
Minocycline (≤4, ≥16)	*P. pabuli*	13	≤0.016 to 0.12	≤0.016	0.12	13 (100)	—	—
	*P. provencensis*	11	≤0.016 to 0.25	0.12	0.12	11 (100)	—	—
	*P. phoenicis*	9	≤0.016 to 0.06	≤0.016	0.03	9 (100)	—	—
	*P. lautus*	8	1.5 to 48	8	32	3 (37.5)	1 (12.5)	4 (50)
	*P. amylolyticus*	6	0.03 to 0.25	0.03	0.25	6 (100)	—	—
	*P. glucanolyticus*	6	8 to 32	8	32	—	3 (50)	3 (50)
	*P. macerans*	6	0.03 to 0.25	0.06	0.25	6 (100)	—	—
	*P. ginsengihumi*	5	≤0.016 to 0.12	≤0.016	0.12	5 (100)	—	—
	*P. thiaminolyticus*	5	0.06 to 32	12	32	3 (60)	—	2 (40)
	*P. timonensis*	5	0.03 to 0.12	0.06	0.12	4 (80)	—	—
Gentamicin (≤4, ≥16)	*P. pabuli*	13	0.25 to 2	0.25	0.5	13 (100)	—	—
	*P. provencensis*	11	0.12 to 1	0.25	1	11 (100)	—	—
	*P. phoenicis*	9	0.25 to 2	0.5	1.5	9 (100)	—	—
	*P. lautus*	8	1 to 4	2	2	8 (100)	—	—
	*P. amylolyticus*	6	0.25 to 0.5	0.25	0.5	6 (100)	—	—
	*P. glucanolyticus*	6	0.12 to 1	0.25	1	6 (100)	—	—
	*P. macerans*	6	0.25 to 3	0.5	3	6 (100)	—	—
	*P. ginsengihumi*	5	0.5 to 1.5	1	1.5	5 (100)	—	—
	*P. thiaminolyticus*	5	0.25 to 4	1	4	5 (100)	—	—
	*P. timonensis*	5	0.25 to >256	16	64	4 (80)	—	1 (20)
Rifampicin (≤1, ≥4)	*P. pabuli*	13	0.25 to 1	0.25	1	13 (100)	—	—
	*P. provencensis*	11	0.06 to 0.25	0.12	0.25	11 (100)	—	—
	*P. phoenicis*	9	0.003 to 0.25	0.003	0.25	9 (100)	—	—
	*P. lautus*	8	0.5 to >32	>32	>32	4 (50)	—	4 (50)
	*P. amylolyticus*	6	0.5 to 12	12	12	2 (33.3)	3 (50)	1 (16.7)
	*P. glucanolyticus*	6	0.03 to 16	16	16	1 (17)	—	5 (83)
	*P. macerans*	6	0.016 to 1	1	1	6 (100)	—	—
	*P. ginsengihumi*	5	0.03 to 1	1	1	5 (100)	—	—
	*P. thiaminolyticus*	5	0.016 to 0.12	0.12	0.12	5 (100)	—	—
	*P. timonensis*	5	0.5 to 2	2	2	4 (80)	1 (20)	—
Cotrimoxazole (≤2, ≥4)	*P. pabuli*	13	>32	>32	>32	—	—	13 (100)
	*P. provencensis*	11	16 to >32	>32	>32	—	—	11 (100)
	*P. phoenicis*	9	0.08 to 0.25	0.03	0.25	9 (100)	—	—
	*P. lautus*	8	0.25 to >32	>32	>32	3 (37.5)	—	5 (62.5)
	*P. amylolyticus*	6	8 to >32	>	>32		—	6 (100)
	*P. glucanolyticus*	6	0.016 to 0.25	0.12	0.25	6 (17)	—	—
	*P. macerans*	6	0.06 to >32	0.25	>32	4 (67)	—	2 (33)
	*P. ginsengihumi*	5	0.12 to 0.5	0.12	0.5	5 (100)	—	—
	*P. thiaminolyticus*	5	0.25 to 1	0.5	1	5 (100)	—	—
	*P. timonensis*	5	0.008 to 0.03	0.03	0.03	5 (100)	—	—
Vancomycin *(*≤4, >4)	*P. pabuli*	13	2 to 4	3	4	13 (100)	—	—
	*P. provencensis*	11	0.03 to 25	2	4	10 (91)	—	1 (9)
	*P. phoenicis*	9	0.38 to 2	0.03	0.25	9 (100)	—	—
	*P. lautus*	8	2 to 8	3	8	6 (75)	—	2 (25)
	*P. amylolyticus*	6	2 to 4	2	4	6 (100)	—	—
	*P. glucanolyticus*	6	2 to 4	2	4	6 (100)	—	—
	*P. macerans*	6	6 to 64	8	64	—	—	6 (100)
	*P. ginsengihumi*	5	1.5 to 2	2	2	5 (100)	—	—
	*P. thiaminolyticus*	5	0.75 to 48	24	48	2 (40)	—	3 (60)
	*P. timonensis*	5	2 to 12	2	12	4 (80)	—	1 (20)

MIC, minimum inhibitory concentration.

a Interpretative criteria of susceptible and resistant breakpoints described for *Bacillus* isolates adopted from Luna et al. [25].

b MICs at which 50 and 90% of the isolates were inhibited, respectively.

Taking into account the number of antimicrobial affected, 33 isolates (24.3%) belonging to 14 species were susceptible to all antimicrobial agents studied except ampicillin. This event is highlighted for *P. phoenicis* (9/9), *P. ginsengihumi* (5/5), *P. thiaminolyticus* (3/5) and *P. timonensis* (3/5), as well as for the *‘Candidatus’ P. infantis, P. mageritense* and *P. vini.* Only nine of 136 of isolates belonging to five species—*P. lautus* (4/8), *P. cineris* (2/2), *P. campinansensis* (1/1), *P. pueri* (1/1) and *P. residui* (1/1)—were resistant to four or more antibiotics.

## Discussion

As early as 1989, the involvement of *P. alvei* in bacteraemia was reported [3]. Since then the number of descriptions of clinical infections caused by the environmental bacteria *Paenibacillus* has increased [4], [5], [6], [7], [8], [9], [10], [11], [12], [13], [14], [15], [16], [17], [18], [19], [20]. However, to our knowledge, literature regarding prevalence of these microorganisms in humans is limited. This may be because in many laboratories they are considered to be contaminants of clinical specimens, even though some are truly pathogens [7], [8], [9], [11], [15], [19], [20]. Further, the staining characteristics of these microorganisms makes the presumptive identification the isolates difficult, as very few isolates are submitted as *Paenibacillus* spp.

The 16S rRNA gene sequencing is the reference standard for identification of *Paenibacillus.* However, it is not enough in some cases, which require phenotypic tests to differentiate closely related species. This occurred for *P. pabuli* from other nearby species such as *P. xylanolyticus, P. tundrae, P. taichungensis* and *P. xylanexedens,* or for *P. cineris* and its nearby species *P. rhizospherae* and *P. flavisporus.*

Between 1999 to 2015, we identified 136 strains of *Paenibacillus* submitted to our reference laboratory for identification from both human and environmental origins. These isolates belonged to 37 different species. Thirty-one were isolated from human samples. Of the 22 species previously isolated from clinical samples [3], [4], [5], [6], [7], [8], [9], [10], [11], [12], [13], [14], [15], [16], [17], [18], [19], [21], we also found ten in human samples, and in some cases in environmental samples. In addition, the presence of 23 species not previously described as having a human origin was found.

We thus can say that the number of species found in humans would be 45 out of a total 211 described species of the genus *Paenibacillus.* To this number must be added the 10 *‘Candidatus’* new species indicated in Table 1. The number of species found in clinical samples would thus rise to 55.

The prevalent *Paenibacillus* species—*P. lautus* and *P. provencensis*—had previously been isolated from human samples [8], [13], whereas *P. pabuli* and *P. phoenicis* had only been isolated from the environment [26]. Our findings are novel in that we identified nine *P. phoenicis* isolates from human sterile sites, eight blood and one cerebrospinal fluid, from four laboratories from different geographic locations and over several years. We found this especially interesting because of the remarkable information available for this species: it was isolated from samples from a molybdenum mine and from environmental controls at the Jet Propulsion Laboratory Spacecraft Assembly facility in Pasadena, California, USA [26]. *P. barengoltzii* was also identified in the same laboratory [26], [27]. Further, we identified three isolates of this species from two ascitic fluid samples and one synovial fluid sample. These are two clear examples extendable to many other species of the genus thanks to its ubiquity, unexplored until now.

The crucial question here is the implication of some of these species of *Paenibacillus* as a true pathogen. In previous descriptions of other species isolated from clinical specimens, the clinical significance could not be determined because no signs or symptoms of serious infection developed in the patients. Thus, in many cases, they were considered to be sample contaminants. Especially in the case of strains isolated from blood, except in cases of proven immunosuppression or other patient-related states, it has been considered to be an asymptomatic contamination [8], [10], [13]. However, in other cases, the isolates are involved in a true infection when the requirements of true bacteraemia are met [28]. Without a doubt, cerebrospinal fluid, wound exudation and abscess isolates caused the infection [3], [7], [9], [15].

Keeping in mind the difficulties of assigning a pathogenic or contaminant role to the isolates, we think that nearly 75% of cases would be contaminants related to the different stages of human sample's management. This abundance of contaminants in human isolates could be explained by our data on isolates recovered from several types of environmental samples: 18 of 48 isolates were collected from workers' gloves in different laboratories, as well as seven other isolates from air and surfaces of the same laboratories. In addition, on six occasions, they were found in laboratory areas where it is performed the control of sterility of biological products and medicines, which could give rise to sources of infection. However, in 25% of the isolates, they were involved in the infectious processes, especially in cases that included abscesses, wound exudates, ocular infections and diverse fluids.

These rules provide more information about isolates in cases of infection with different organisms and with different involved sites. Several virulence markers have recently been found in some species of *Paenibacillus,* such as *P. lautus* and *P. amylolyticus* [29]. Those data, together with new research on the existence of virulence genes in other species isolated from clinical samples, will shed light on the dilemma on the pathogenic vs. merely opportunistic and contaminating role of these species in humans.

Finally, we would like to point out the polyphasic taxonomic approach of 11 *‘Candidatus’* to new species of the genus, ten of them isolated from clinical samples, which imply its abundance, along with the 28 new species described in 2016 (with isolates from soil, human faeces, yak milk, termites, plants and *Rhizosphaera*)—a remarkable increase in the number of species of *Paenibacillus* and a marker of its ubiquity.
